# The New Sydenham Society

**Published:** 1867-08

**Authors:** 


THE NEW SYDENHAM SOCIETY.—The aims of this Society are best set forth in the following extract from their laws:

I. "The Society is instituted for the purpose of supplying certain acknowledged deficiences in the existing means of diffusing medical literature, and shall be called The New Sydenham Society.

II. "The Society shall carry out its objects by a succession of publications, of which the following shall be the chief: 1. Translations of Foreign Works, Papers, and Essays of merit, to be reproduced as early as practicable after their original issue. 2. British Works, Papers, Lectures, etc., which, whilst of great value, have become from any cause difficult to be obtained, excluding those of living authors. 3. Annual Volumes, consisting of Reports in Abstract of the progress of different branches of Medical and Surgical Science during the year. 4. Dictionaries of Medical Bibliography and Biography. Those included under Nos. 1 and 2 shall be held to have the first claim on the attention of the Society, and the carrying out of those under 3 and 4 shall be considered dependent upon the amount of funds which may be placed at its disposal.

During its brief existence of nine years, this Society has published thirty-five volumes upon various medical subjects, all of which being of practical and permanent value. Prominent amongt these publications is the Society's Atlas of Portraits of Skin Diseases, taken from the plates of the celebrated dermitologist, Dr. Hebra. The annual subscription price of one guinea, or seven and a half dollars, entitles the subscriber to the series of volumes published during the year; the entire series or any part thereof can be procured. All communications should be addressed to the Honorary Secretary Dr. Dunglison, 1116 Girard Street, Philadelphia. The following list of works constitute the series for 1866 and 1867:

SERIES FOR 1866.

1. Bernutz and Goupil on Diseases of Women. Vol. 1.
2. Fasciculus of Atlas of Portraits of Diseases of the Skin (three beautiful colored plates, life size).
3. Hebra on Diseases of the Skin. Vol. 1.
4. Bernutz and Goupil on Diseases of Women. Vol. 2.

SERIES FOR 1867.

1. Griesinger on Mental Diseases.
2. Biennial Retrospect of Medicine and Surgery.
3. Fasciculus of Atlas of Portraits of Diseases of the Skin (colored plates).
4. Hebra on Disease of the Skin. Vol. 2.

---

THE nmerous friends of Surgeon J. H. Baxter gained by valuable services during the late war, will be pleased to learn of his advancement to the rank of Lieutenant Colonel. We know of no promotion in the Medical Corps of the Army more deservedly earned and giving more general satisfaction than that of our friend, and we would tender him our sincerest congratulations. The following paragraph we clip from the Washington *Chronicle:*

CONFIRMED.—We are pleased to notice among the confirmations by the Senate on Saturday, that of Surgeon J. H. Baxter, U. S. Vols., late chief medical officer of the Provost Marshal General's Bureau, as assistant medical purveyor, U. S. A., with the rank of Lieut. Colonel. Dr. Baxter served with great credit during the entire war, and this mark of appreciation of his services by the Surgeon General, Secretary of War, the President, and the unanimous voice of the Senate, must be peculiarly gratifying to his friends and to the late surgeons of the boards of enrollment throughout the United States. This promotion will not interfere with the completion of the medical report of the Provost Marshal General's Bureau, upon which Dr. Baxter is now engaged, in accordance with a resolution of Congress.

We are in receipt of the following Circular from George Mendenhall, M. D., Chairman of a Committee on Medical Literature, appointed by the American Medical Association. We hope that each of our readers will render any aid in their power to make the report of the Committee as complete as possible:

CINCINNATI, June 5th, 1867.

The undersigned were appointed at the last annual meeting of the American Medical Association, held in Cincinnati, a Committee on Medical Literature for the current year. The duties of this Committee are defined in the following regulations of the Association:

"The Committee on Medical Literature shall prepare an annual report on the general character of the periodical medical publications of the United States with reference to the more important articles therein presented to the profession, on original medical publications, on medical compilations and compends by American writers, on medical reprints of foreign medical works; and on all such measures as may be deemed advisable for encouraging a national literature of our own."

Being desirous of making as full a report as possible, the Committee desire that you shall forward to the Chairman a copy of all medical books, pamphlets, essays, monographs, periodicals, reports, lectures, proceedings of societies, etc., that may be issued by you, as early as convenient after publication, that they may be brought to the notice of the profession.

These favors will be advantageous to publishers, and will facilitate the objects had in view by the appointment of the Committee, and greatly oblige,

Yours, respectfully,
GEO. MENDENHALL, Chairman.
R. R. MCILVAIN, GEO. C. BLACKMAN,
E. WILLIAMS, P. S. CONNOR.

